# Interactions between ectomycorrhizal fungi and chestnut blight (*Cryphonectria parasitica*) on American chestnut (*Castanea dentata*) used in coal mine restoration

**DOI:** 10.3934/microbiol.2018.1.104

**Published:** 2018-02-11

**Authors:** Jenise M. Bauman, Sarah Francino, Amy Santas

**Affiliations:** 1 Western Washington University, Huxley College of the Environment, Bellingham, Washington, 98225, USA; 2 Muskingum University, 163 Stormont Street, New Concord, Ohio, 43762, USA

**Keywords:** reforestation, restoration, plant and fungal interactions, ectomycorrhizal community development, American chestnut restoration, disease die-back

## Abstract

Plant and fungal interactions drive successional trajectories within reforestation offering both mutualisms (ectomycorrhizal fungi [ECM]) and fungal pathogens. Appalachian forest and mine reclamation projects re-introducing American chestnut and chestnut hybrids will inevitably document the return of chestnut blight, resulting in cankers causing branch dieback and loss of photosynthetic tissue. Similar to herbivory, the loss of photosynthetic tissue may reduce ECM root colonization and cause changes in fungal species composition. To test this, 75 six-year-old established chestnut trees were selected to represent the following: (1) Healthy trees free of chestnut blight; (2) trees with cankers and 50% branch dieback; (3) trees that died prior to the fifth growing season. Each tree had a chestnut seed planted 24 cm from the base. ECM colonization of both the established parent trees (n = 50) and five-month-old seedlings (n = 64) were quantified and genera determined by fungal DNA sequencing of the internal transcribed (ITS) region. Healthier seven-year-old chestnuts trees had significantly more ECM roots than those trees infected with chestnut blight cankers. However, disease die-back on chestnut did not have an influence on community composition among the parent trees or the neighboring five month seedlings. Results also demonstrated that five-month-old seedlings neighboring healthy parent trees had greater ECM on roots (*P* = 0.002), were larger in size (*P* = 0.04), and had greater survival (*P* = 0.01). ECM genera such as *Cortinarius*, *Russula* and *Scleroderma* provided tree to seedling inoculation. ECM colonization by *Cortinarius* spp. resulted in larger chestnut plants and increased nitrogen foliar concentrations on the five month seedlings. It can be hypothesized that blight will aid in diversifying forest stand composition and these early ECM networks will help facilitate the survival of other native hardwoods that recruit into these sites over time.

## 1. Introduction

Plant and fungal interactions drive successional trajectories and community assembly within reforestation. Mycorrhizal fungi create symbiotic relationships with tree roots and facilitate the transfer of water and nutrients in exchange for photosynthates [1]. Plant co-existence may be facilitated by various mycorrhizal associations, with specific symbionts providing access to limiting resources as succession progresses [2],[3]. The development of common mycorrhizal networks (CMN) can facilitate seedling recruitment by shared nutrient transfer from plant to plant via source to sink, aiding in co-existence within guilds [2],[4],[5]. Fungal pathogens also contribute to structuring plant communities by driving species above- and belowground turnover as plant succession progresses [6],[7]. Numerous studies have documented increased enhanced stability with diversity and increased community productivity and biomass due to density dependent pathogen mortality [8]–[10]. As natural disturbances, root and wood rots maintain gap dynamics in forests contributing to resource availability, productivity and forest structure [11],[12].

Large-scale canopy shifts have been demonstrated with the introduction of chestnut blight (*Cryphonectria parasitica* [Murr.] Barr), a non-native and invasive fungal pathogen introduced in late 1800's from nursery stock of Japanese and Chinese chestnut [13]. The fungus enters through wounds in bark and produces cankers that kill cells within the tree's cambium region through the action of enzymes produced by the invading mycelial fans [14]. This fungal pathogen is highly specific to the American chestnut (*Castanea dentata* [Marsh.] Borkh), which has no natural resistance. This highly virulent pathogen and prolific tree host led to rapid disease spread and complete extirpation of a keystone tree species resulting in a significant canopy shift [15]. Culturally, this species represented an important agro-ecological role that cultivated both timber, nut crops and tannin production in the Appalachian region, while providing a habitat for biodiversity throughout North American forests [16]. Chestnut's historical relevance and once ecological stature has inspired breeding programs that eventually led to backcrossing methodology initiated in the 1980s, which have produced American chestnut hybrids with putative resistance to chestnut blight [16],[17]. Experimental plantings in forests [18]–[22] and afforestation projects on reclamation lands [23]–[27] have facilitated the introduction of backcrossed chestnut hybrids into the native range of American chestnut in North American forests [28].

However, the native soil community can be a limiting factor in the restoration and establishment of native trees [2]. American chestnut is an ectomycorrhizal (ECM) host [29]–[31] and its return to North American forests can be facilitated by soils that harbor locally adapted and chestnut compatible ECM species associated with the oak forests [32]. However, on mine reclamation sites, drastic soil disturbances led to significant changes in ECM community composition [33]–[35]. To overcome this, seedlings used in reclamation projects are usually pre-inoculated with selected ECM fungi in field nurseries or in greenhouses prior to tree plantings, which may bypass some soil limitations and accelerate the establishment of mid to late successional species in initial plantings [36],[37]. Further, planting methods that maximize ECM root colonization also report greater chestnut growth and survival in the early years of chestnut restoration [38],[39].

Appalachian forest and mine reclamation projects re-introducing American chestnut and chestnut hybrids will inevitably document the return of chestnut blight [21],[25]. Cankers resulting in branch dieback and loss of photosynthetic tissue may deter the host's ability to maintain root colonization by ECM fungi. Similar to herbivory, the loss of photosynthetic tissue may reduce ECM root colonization [40],[41] and cause changes in fungal species composition [42]. However, not all studies documented a change in ECM colonization after artificial herbivory [43],[44] or a shift in ECM community composition due to tissue loss [45]. Further, the influence of *Phytophthora* root pathogens resulting in defoliation of forest trees has also reported mixed results from significant reduction in ECM associated with *Eucalyptus* crown [46], to no change in ECM community as a response to defoliation on *Quercus* [47]. Blom et al. [48] reported no relationship between a declining sweet chestnut (*C. sativa*) due to *Phytophthora* and ECM colonization, however, they reported significantly finer ECM hyphal morphologies in healthy plots, presumably due to reduced photosynthetic capacity of its host. Therefore, ECM had a more robust morphotype when associated with healthy trees, which was similar to significant shifts in fungal morphotypes with tissue loss favoring thin hyphal mantles [49],[50].

American chestnut trees can be hypothesized to display a similar pattern; the presence of chestnut blight in restoration stands would decrease ECM colonization on roots and shift ECM morphologies, with healthy trees selecting fungal species that display thicker mantels and radiating hyphal cords, and trees with dieback selecting fungi that display thinner hyphal morphologies. The objective of this current study is to document ECM root colonization and community composition on healthy and declining chestnut trees caused by chestnut blight, document inoculum potential of a neighboring chestnut tree in various stages of disease, and identify beneficial attributes of fungal species with regard to nitrogen acquisition. To test this, American chestnuts were categorized based on three various stages of health: (1) Chestnut trees free of chestnut blight cankers and exhibiting vigorous growth; (2) chestnut trees with chestnut blight cankers caused by *C. parasitica* and exhibiting approximately 50% dieback; (3) seedlings that were recorded as dead in the field after three growing seasons. We hypothesized that loss of photosynthetic tissue will have an influence on ECM root colonization and the availability of reservoir inoculum available for new seedlings sown as nuts in the field. Further, we predict that plant die-back will drive the selection of finer hyphal species as well as decrease the amount of inoculum available to introduced, germinating chestnut seedlings.

## 2. Materials and methods

### 2.1. Study site

The field site is located in the Tri-Valley Wildlife Management Area (TVWMA), Muskingum County, Ohio, USA (40° 6′ 44″ N, −81° 58′ 23″ W). This coal surface mine site was excavated in the 1960–70s and reclaimed in 1978. Reclamation included reconstructing and compacting slopes (6%–20%) using rock overburden and coal mine spoil. Slopes where then covered with the original soil that had been excavated and stockpiled prior to surface mine operations to a depth of 45 cm and regraded to match contour. To prevent soil erosion after grading, slopes were seeded with birdsfoot-trefoil (*Lotus corniculatus*), tall fescue (*Festuca* *arundinacea*), orchard grass (*Dactylis glomerata*), alfalfa (*Medicago sativa*), red clover (*Trifolium pratense*), rye grass (*Lolium perenne*), timothy grass (*Phleum pratense*), Kentucky blue grass (*Poa pratensis*) and Chinese lespedeza (*Lespedeza cuneata*).

For this study, chestnut seeds were sown in March 2006 at the State Nursery in Marietta, Ohio by the Ohio Department of Natural Resources. Seedlings were germinated in seed beds with soils that were injected with spores of mycorrhizal fungus, *Pisolithus tinctorius*, were field nursery grown for one year, and then lifted as bare root seedlings and planted in the field site in March of 2007. The study site was prepared for planting by the installation of three experimental plots, each 18 × 36 m, and cross-ripped to a 1 m depth on 2.15 × 2.15 m grid, followed by plow and disking to 30 cm. At the time of planting soil pH ranged from 5.4 to 5.7 and the soil texture averaged 61% sand, 23% silt and 16% clay. Mean values for soil nutrients were: Aluminium, 3.5 ppm; calcium, 720 ppm; potassium, 78 ppm; magnesium, 182 ppm; manganese, 3.75 ppm; nitrogen, 2 ppm; phosphorus, 8 ppm [38]. When trees were sampled for ECM, surrounding vegetation was primarily tall fescue (*F. arundinacea*; 29%), Chinese lespedeza (*L. cuneata*; 16%), Canada golden rod (*Solidago Canadensis*; 11%) and black-eye Susan (*Rudbeckia hirta*; 11%).

In each of the three plots, 100 chestnut seedlings were planted at 2.15 m spacing as bare-root seedlings within 48 hours after seedlings were lifted from the field nursery (300 chestnut bare rooted seedlings total). Genetic seedling lines represented pure American chestnuts (*C.* *dentata*) and two *C.* *dentata* and *C.* *mollissima* backcrossed chestnuts (BC_1_F_3_ and BC_2_F_3_) predicted to average 75% and 88% inheritance from the American chestnut parent, respectively [17]. To buffer against root desiccation, the root system of each seedling was dipped in TerraSorb gel prior to planting. Two fertilizer pellets (20-10-5) were put in each hole and the seedling was backfilled with original soil. A 1 m × 1 m weed mat was used around each seedling to prevent competition from regenerating herbaceous plant species. Also, a 1.5-m tall chicken wire cage was installed to prevent browse.

To assess the influence disease die-back has on seedling recruitment, 75 seven-year-old, established chestnut trees representing three distinct parent trees were designated: (1) Seven-year-old, were considered healthy chestnut parent trees with no sign of disease and averaging 240 cm in height and 4.0 cm in basal diameter (Table 2); (2) seven-year-old chestnut tree exhibiting dieback (over 50%) and signs of canker caused by *C. parasitica* with average heights of 132 cm with basal diameters averaging 2.4 cm (Table 2); (3) a chestnut seedling with complete main stem mortality. These seedlings were recorded as dead after five field seasons with no re-sprouting. Dead trees averaged 62 cm in height with 0.9 cm basal diameters. The cause of mortality could not be determined. These designations formed the basis of the three treatment groups referred to as “parent trees” (healthy, diseased, dead), each with 25 replications of each treatment group.

To test the influence that the health of a neighboring tree has on establishing seedlings, a nut was planted at the base of each parent tree. The chestnut seeds were provided to us by the American Chestnut Foundation. Nuts were surfaced sterilized using 10% bleach solution for 10 minutes, rinsed with DI water, and allowed to air dry prior to being cold-stratified in peat at 2 °C for 6 weeks. At the time of planting in March, vegetation was cut-back, and a hole approximately 12 × 12 cm was prepared, due south of the dripline of each established tree (approximately 35–45 cm from the trunk). Nuts were field germinated, allowed to grow for 5 months, and harvested (described below). These seedlings will be herein referred to as “five-month seedlings”.

At the time of planting, soils were sampled by extracting soil cores using a soil probe to a depth of 18 cm. Three cores were sampled from the rhizosphere per parent tree. Soil samples were oven dried for 72 hours at 105 °C and sent to Spectrum Analytic Inc., Washington Court House, Ohio, for Mehlich 3 extract analysis. Soil parameters analyzed included: pH, cation exchange capacity (CEC), phosphorus (P), potassium (K), magnesium (Mg), calcium (Ca), sulfur (S), boron (B), zinc (Zn), iron (Fe), copper (Cu) and manganese (Mn).

### 2.2. Sampling for ECM fungi: Five-month seedlings

All surviving five-month-old seedlings, representing each parent tree disease status (n = 64), were carefully removed from the soil as whole seedlings and returned to the laboratory where root systems were washed with DI water and cut into approximately 3 cm segments. One hundred root tips per seedling, where applicable, were randomly selected from each seedling, viewed under a dissecting microscope for the presence of a fungal sheath and irradiating hyphae. Using a grid template as a guide, the number of ECM root tips was divided by the total number if roots sampled to calculate percent ECM. ECM was morphotyped based on their color and texture of sheath, emanating hyphae and presence of rhizomorphs [38]. Authors did not specify dead verses living root tips and only scored root tips as ECM or non-ECM. At the time of sampling in 2013, all seedling recruits were measured for height (cm), basal diameter (mm) and volume by dry weight (g).

### 2.3. Foliar nitrogen analysis: Five-month-old-seedling

Leaves from all 5-month-old seedlings were removed, oven-dried at 80 °C for 72 hours and then homogenized using a mortar and pestle. Nitrogen (N) was analyzed using a Thermo Electron NC Soil Analyzer Flash EA 1112 Series (Western Washington University, Department of Environmental Science). Dried leaf tissue weighing approximately 20 mg were placed in tin capsules and compressed to remove any air prior to carbon-nitrogen analysis. The Eager 300 Software (Thermo Electron S.p.a., Milan, Italy) provided a chromatogram graph and percent composition of total carbon and nitrogen.

### 2.4. Sampling for ECM fungi: Chestnut parent trees

All parent trees (25 healthy and 25 diseased) were selected for non-destructive sampling for ECM fungi at this time. To sample for ECM fungi, three soil cores (10 cm × 10 cm) were collected from the drip line of each 7-years old chestnut tree. Samples were pooled among cores, per seedling, stored on ice, and returned to the laboratory within 24 hours. In the laboratory, soil cores were sifted using mesh screen, roots were washed with DI water, and transferred into a Petri dish containing sterile water. One hundred root tips per tree were randomly selected from each tree and viewed under a dissecting microscope for the presence of a fungal sheath (Table 1). Each ECM tip was sorted into one of the nine morphotypes based on their surface color, texture, emanating hyphae, and rhizomorphs based on morphologies illustrated in Bauman et al. [38]. ECM root tips that did not match previous morphologies were described, designated as new morphotypes, and assigned a new NCBI accession number.

### 2.5. DNA extraction and sequencing

Presence and type of ECM species on the root tip was determined by DNA extraction followed by PCR amplification and DNA sequencing of the ITS region of the fungal genome. For each seedling, a 3-mm segment of root tip was removed, transferred to a microcentrifuge tube, and stored at −70 °C until DNA was extracted. Two samples per morphotype were selected. Root tips were homogenized using a bead beater and DNA was extracted using Qiagen's DNeasy Plant Mini Kit (Qiagen, Hilden, Germany) per manufacturers protocol. About 10 ng of this DNA was used in PCR reactions using primers ITS1-F (5′-CTTGGTCATTTAGAGGAAGTAA-3′) and ITS4 (5′-TCCTCCGCTTATTGATATGC-3′), which amplify the highly variable internal transcribed spacer (ITS) region of ECM fungal ribosomal DNA (rDNA). The PCR products were purified using Wizard^®^ SV 96 Genomic DNA Purification System (Promega, Madison, WI, USA). DNA was sequenced by capillary Sanger sequencing at the Plant-Microbe Genomics Facility of The Ohio State University (Columbus, OH, USA). The DNA sequences were edited by trimming low quality reads off and visually inspecting and editing chromatograms from contigs using Sequencher 4.2 software (Gene Codes, Ann Arbor, Michigan). By comparing these sequences to those present in the GenBank and UNITE database, identity of the ECM fungus was determined [51],[52]. To be conservative, we only reported the sequences that did match >95% of the data base. Therefore, authors acknowledge that other species of genera reported may have been sampled, however, lacked the similarity match to be considered an additional species within the genera reported in this paper. For those sequences that matched authors previously reported ECM morphotypes on chestnut (>98% similarity), the original accession is reported (Table 1). For new ECM taxon, authors deposited sequences into GenBank and reported the new accession numbers.

### 2.6. Statistical analysis

ECM colonization per treatment was assessed by taking the percentage (#ECM tips/100) of ECM colonized root tips from parent trees and neighboring five-month seedlings. Description of ECM per site was quantified by species richness and Shannon-Weiner diversity index based on ECM tip counts for each morphotype. Two sample t-tests were used to detect significant differences between healthy and diseased chestnuts with regard to ECM root colonization, species richness and diversity. An ANCOVA followed by Tukey's HSD post hoc was used to assess differences among parent trees (main effect) to seedling ECM colonization, number of fungal species and seedling height (dependent variables). Means are reported ± standard error (SE). Arcsine and log transformations were used, when needed, to meet the assumption of similar variance and normal distribution. To compare seedling survival among parent trees, a Pearson's Chi-Square (*X^2^*) was used. Pearson correlation analysis was used to compare the strength of relationships between foliar nitrogen concentrations and two ECM root colonization groups: (1) those that were colonized by *Cortinarius* spp.; (2) those colonized by non-*Cortinarius* species such as *Inocybe*, *Scleroderma*, *Hebeloma* and *Cenococcum*. All statistical tests were performed using R v2.91 [53].

All following community statistics were analyzed using Vegan: Community Ecology Package, version 2.3.0. Overall species richness of the ECM community was estimated using two common incidence based richness estimators, Chao II and a bootstrap population estimator by the “specpool” function. A non-metric multidimensional scaling (NMDS) ordination and a Bray-Curtis dissimilarity matrix was used to describe ECM species composition based on root tip counts between parent trees and five month seedlings, and ECM composition among the disease classifications (healthy, diseased and dead) using “metaMDS” function. To improve the NMDS ordination, rare species that scored <0.5% based on root tip counts were removed from the data set and data were square-root transformed and standardized using Wisconsin double standardization. The analysis ran for 100 random starts until the best solution, decided by the lowest stress value, was reached.

A two-way permutational multivariate analysis of variance as used to test for significant differences among the parent trees, five month seedlings, disease classifications (healthy, diseased, dead), and their interaction using the “adonis” function in Vegan. This method partitions variation in dissimilarity or distance matrices using a “pseudo-F” statistic analogous to MANOVA [54]. The correlation of soil chemistry (CEC, pH, P, K, Mg, Ca, CEC, S, B, Cu, Fe, Mn, Zn), organic matter, tree basal diameter (cm) and tree height with NMDS axes (cm) were analyzed using Mantel tests, which are used to test for correlations between two matrices [54]. Soil variables were standardized and fit onto the NMDS and squared correlation coefficients and *P*-values were determined by the “envfit” function in R [53],[54].

## 3. Results

### 3.1. ECM community composition

Through DNA sequencing, 17 fungal species were documented in ectomycorrhizal association with 7-year-old chestnut trees and 5-month-old chestnut seedlings (Table 1). Sampling efficacy was verified using Chao II and bootstrap richness estimators, which reported 19 ± 3.3 and 18 ± 1.2 ECM species, respectively. A two-way permutational ANOVA on the ECM community matrices showed that differences in ECM communities existed between the two chestnut types (parent trees verses five-month seedlings; *F*_(1,10)_ = 2.33, *P* = 0.03). Differences in ECM community composition did not exist by disease status of the chestnut trees or seedlings. Further, no interactions between tree age and disease status existed. Therefore, ECM species are presented by chestnut type (parent tree verses five-month seedlings) in Table 1.

**Figure 1.**
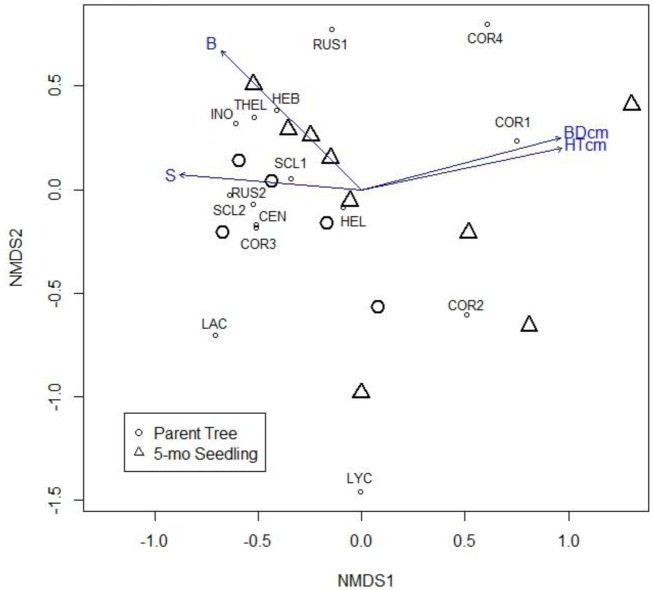
Non-metric multidimensional scaling (NMDS) ordination (stress = 0.13) of ECM fungi based on American chestnut root tip counts. Larger circles (○) symbolize seven-year-old parent trees and triangles (Δ) symbolize the five-month-old seedlings (5-mo seedlings). Small circles (∘) represent ECM species values with species' names appearing as abbreviations on the ordination (see Table 1). The pattern reveals that ECM communities differed between the two chestnut age groups (permutational multivariate analysis of variance: *P* = 0.03). Plot vectors indicate strength and direction of the strongest correlations between plant measurement and soil variables and with regard to ECM species detected. Chestnut growth (height and basal diameter), as well as soil sulfur (S) and soil boron (B) concentrations significantly influenced ECM community composition.

Both the trees and seedlings shared the most abundant species *Cortinarius* *subbalaustinus* (COR1). This species was strongly associated with the first NMDS1 axis of the ordination, and was significantly correlated to seedling height (r = 0.50, *P* = 0.01) and basal diameter (r = 0.52, *P* = 0.009; Figure 1). *Scleroderma* *areolatum* (SCL1) and *Russula* *pectinatoides* (RUS1) were also shared between both the 7-year-old parent trees and 5-month-old seedlings (Table 1). However, certain ECM species such as *Cenococcum* sp. (CEN), *C. parvannulatus* (COR3), *R.* *cerolens* (RUS2) and *S.* *citrinum* (SCL2) were highly associated with the parent trees, which appeared in the ordination negatively correlated with sulfur (S) concentrations in the soil (r = 0.39, *P* = 0.04; Figure 1). In contrast, species such as *C.* *decipiens* (COR2), *Hebeloma* sp. (HEB), *Inocybe* *rufoalba* (INO) and *Thelephora* *terrestris* (THEL) were associated with the five- month seedlings and positively correlated to soil concentrations of boron (B; r = 0.39, *P* = 0.04). Species such as *C. diasemospermus* (COR4) and *Laccaria* sp. (LAC) were not commonly sampled and therefore, difficult to determine with regard to the preference of host age. Other ECM included an unknown species as well as matching sequences in the order *Helotiales*, *Lactarius* sp. and *Oidiodendron* sp. were sampled in trace amounts (<0.5%) and therefore were not included in the NMDS (Table 1).

**Table 1.** Ectomycorrhizal (ECM) fungal species abundance generated from root tip count data from chestnut roots from two sources: Seven-year-old parent chestnut trees (Parent Trees, n = 50) and the five-month seedlings (n = 64). ECM species were matched to both UNITE and NCBI data base and are listed alphabetically along with their abbreviation used in the NMDS ordination (illustrated in Figure 1) and corresponding NCBI Accession number. Asterisk (*) indicates a match from a previous submission.

ECM Matched	NMDS	Parent Tree	5-month-seedling	Accession #
Unknown 1	N/A	0.1	0	N/A
*Cenococcum geophilum*	CEN	23.4	7.0	GU246995*
*Cortinarius subbalaustinus*	COR1	41.0	38.6	GU246986*
*Cortinarius decipiens*	COR2	3.4	9.8	GU246987*
*Cortinarius parvannulatus*	COR3	8.1	1.2	MG674533
*Cortinarius diasemospermus*	COR4	2.1	0	MG674534
*Hebeloma* sp.	HEB	0	6.0	MG674535
*Helotiales*	HEL	0	0.5	MG674536
*Inocybe rufoalba*	INO	1.6	10.2	MG674537
*Laccaria bicolor*	LAC	0.5	0.1	GU246994*
*Lactarius chrysorrheus*	N/A	0.3	0	MG674538
*Oidiodendron maius*	N/A	0	0.4	MG674540
*Russula pectinatoides*	RUS1	3.7	3.7	MG674541
*Russula cerolens*	RUS2	2.3	0	MG674542
*Scleroderma areolatum*	SCL1	10.0	12.8	GU246989*
*Scleroderma citrinum*	SCL2	2.2	0.2	GU246990*
*Thelephora terrestris*	THEL	1.5	9.6	GU246993*

**Table 2.** Comparison of tree height (cm), basal diameter (cm), percent (%) ectomycorrhizal fungal (ECM) root colonization, ECM species richness and ECM diversity based on Shannon-Weiner diversity index on seven-year-old parent chestnut trees. Means sharing common letters do not significantly differ at α = 0.05.

Parent Tree	Height (cm)	Basal Dia (cm)	% ECM	ECM Richness	ECM Diversity
Diseased	131.8 ± 7.8^a^	2.4 ± 0.2^a^	52 ± 4.9^a^	4.3 ± 1.9^a^	1.4 ± 0.3^a^
Healthy	239.8 ± 3.2^b^	4.0 ± 0.2^b^	72 ± 3.9^b^	6.0 ± 0.6^a^	1.4 ± 0.1^a^

### 3.2. Disease influence on ECM colonization on parent trees

Seven-year-old healthy parent trees had significantly more root colonization (72%) than parent trees experiencing tissue loss associated with chestnut blight (52%; t = 3.23, df = 23, *P* = 0.002). However, no differences existed when species richness was assessed; both tree categories averaged approximately three species (Table 2), which was similar to the NMDS community composition.

### 3.3. Disease influence on ECM colonization on five-month seedlings

**Figure 2.**
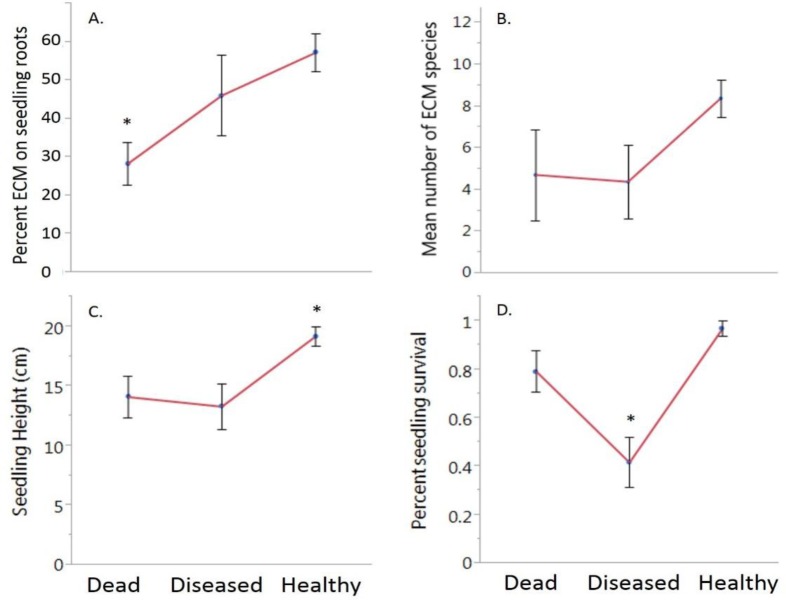
Comparison of ECM root colonization (%), seedling height (cm), and survival of five-month seedlings growing next to dead, diseased, and healthy seven-year-old chestnuts trees growing on a reclaimed surface mine site in eastern OH, U.S.A. Asterisks (*) indicate significant differences at 0.05 alpha. Panel A: Five-month seedlings that were growing among healthy trees had the most ECM roots while seedling recruits growing with the dead trees had the least. Diseased seedlings were intermediate with regard to percent root colonization. Panel B: There were more ECM species documented on five-month chestnut seedlings neighboring healthy trees, however, this was not statistically significant. Panel C: Comparisons of five month seedling height (cm) illustrate larger seedlings were found growing at the base of healthy and dead trees. Diseased trees neighbored significantly smaller seedling. Panel D: Survival was dependent on parent trees, healthy trees had a higher survivorship among the five-month seedlings, followed dead trees and diseased trees. Error bars are ±1 SE.

Differences were significant with regard to ECM root colonization on the five-month-old seedling recruits among the healthy, diseased and dead neighboring trees (F = 6.67, df = 2, *P* = 0.002), with apparent benefits associated with healthy established trees (Figure 2). Seedling recruits growing next to healthy parent chestnut trees had the greatest ECM root colonization (58%), colonization of seedlings growing next to diseased trees was similar (44%). Both healthy and diseased trees had significantly more ECM than seedlings growing adjacent to dead trees (28%; Figure 2A). Though seedling recruits neighboring healthy trees had a greater number of ECM species, this was not significant (Figure 2B). Seedling recruits growing next to healthy trees were significantly taller (19.2 cm) than those growing at the base of diseased (13.3 cm) and dead trees (14.1 cm; F = 6.24, df = 2, *P* = 0.003; Figure 2C). Survival of the five-month-old recruits also differed among the treatments with greater survival of seedlings next to healthy trees (98%) followed by seedlings next to dead trees (80%), with poorest survival when seedling recruits were neighboring diseased trees (42%; *X^2^* = 25.9, *P* < 0.0001; Figure 2D).

### 3.4. Nitrogen acquisition by ECM five month seedlings

**Figure 3.**
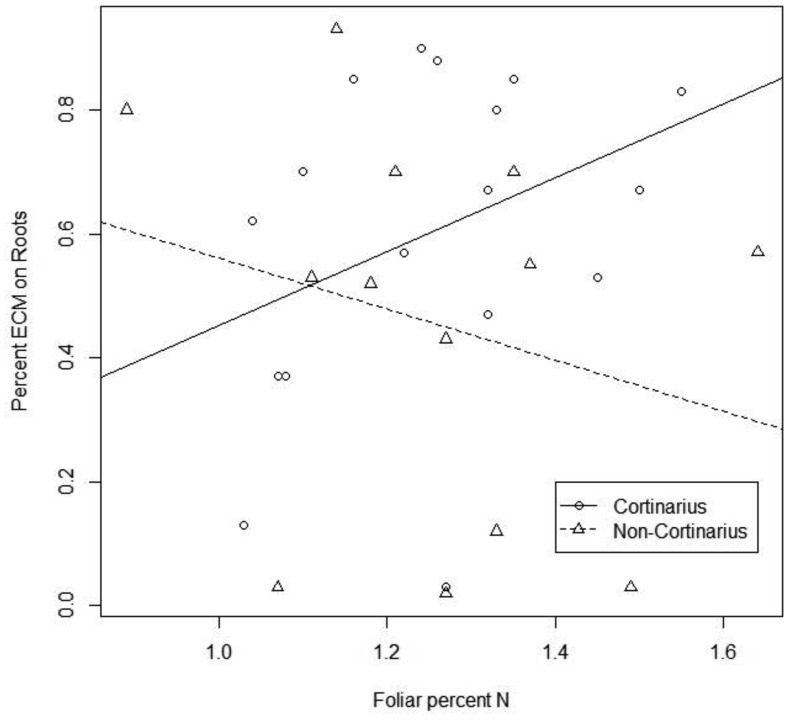
Scatter plot illustrating correlations of nitrogen acquisition (foliar percent N) and percent ECM on roots of five month ECM seedlings was analyzed between seedlings that were colonized by *Cortinarius* spp. (circle with solid line) and those colonized by non-*Cortinarius* species such as *Inocybe*, *Scleroderma*, *Hebeloma* and *Cenococcum* (triangle with broken line). A positive correlation existed between *Cortinarius* ECM colonization and foliar N in seedling tissue (*P* = 0.05).

Nitrogen acquisition of five month ECM seedlings was analyzed between two distinct groups: those that were colonized by *Cortinarius* spp. and those colonized by non-*Cortinarius* species such as *Inocybe*, *Scleroderma*, *Hebeloma* and *Cenococcum*. A positive correlation existed between *Cortinarius* ECM colonization and foliar N in seedling tissue (r = 0.45, t = 2.07, df = 17, *P* = 0.05; Figure 3). However, the non-*Cortinarius* ECM species illustrated a negative trend, but this was not statistically significant (r = −0.31, t = −1.17, df = 13, *P* = 0.26). A significant correlation did not exist when five-month-old seedling height or biomass was compared.

## 4. Discussion

### 4.1. Influence of disease dieback

Healthier seven-year-old chestnuts trees had significantly more ECM root tips than those trees infected with chestnut blight cankers. The decrease in ECM on diseased roots was similar to herbivory studies that documented the loss of photosynthetic tissue associated with loss of ECM colonization [41],[42], which supports the hypothesis that ECM colonization is driven, and maintained by carbon available from American chestnut trees. However, disease die-back on chestnut did not have an influence on community composition between the established trees, or seem to drive the selection of fungal species that were more tolerant of reduced carbon. Luoma and Eberhart [55] noted a decrease in mycorrhizal root tip density and ECM species richness correlated with defoliation due to Swiss needle cast. Other studies report a change in fungal community after herbivory [54], root disease [48] and canopy decline [46],[56]. Blom et al. [48] documented an increase in ECM species richness from chestnuts infected with root rot and speculated that healthy trees are more selective than the diseased trees and diseased trees may allocate more carbon belowground due to root mortality. In our study, to compensate for loss of photosynthetic tissue, it is likely that the tree would re-allocate carbon resources to aboveground growth. Also, our study was conducted on a restoration site with disturbed soils with a small, early-seral ECM community, which are likely to have characteristics such as lower carbon demand and prolific spore dispersal. Therefore, if the same study was done in a mature forest, with many dozens of ECM species, and a larger sample size, the effect of disease may have been more apparent.

Healthy neighboring chestnut trees were also associated with an increase in ECM inoculum available to five-month-old seedlings. Additionally, seedlings neighboring healthy trees grew larger and had the highest survival. Approximately half of the seedlings were inoculated by three *Cortinarius* species, with *C. subbalaustinus* the most abundant on both trees and seedlings, presumably modeling mycelia dispersal from existing roots to germinating seedlings [57],[58]. Inoculation from roots of existing plants is an efficient mechanism for root colonization to an establishing seedling [30],[57]. Also, it has been documented that establishing seedlings can root into a common mycorrhizal network [1], which will allow for carbon and nutrient transfer from healthy parent trees (carbon source) to the newly establishing seedlings (carbon sink) [4].

Although diseased trees were statistically comparable to healthy trees regarding inoculum potential, seedling recruits neighboring diseased trees were significantly smaller and had the lowest survival (56%). In contrast, there was a significant decrease in ECM root colonization on seedlings neighboring dead chestnut trees and adequate survival (80%). However, there was also a significant reduction in height, which may be linked to inadequate root colonization (<50%) that would be required for a significant increase in biomass, as seen from seedlings neighboring healthy trees. Therefore, it appears that radial fungal hyphae and ECM roots surrounding a healthy tree provide immediate and effective inoculum for colonization of a neighboring seedling. Although the seeds planted around dead trees had similar ECM communities, the slight decline in survival and reduction in growth may reflect delayed ECM establishment and diminished benefits to the seedling.

### 4.2. ECM species sampled

*C.* *subbalaustinus* was the most abundant species sampled on both parent trees and five-month seedlings. Three additional *Cortinarius* species were also sampled at this time. Members of this genus are medium distance fringe exploring basidiomycetes that form extensive soil mycelia characterized by dense hydrophobic hyphal cords and rhizomorphs, assumed to be efficient in moving materials over relatively large distances [59]–[62]. *Cortinarius* ECM colonization was positively associated with increased levels of foliar nitrogen in five-month-old seedlings. This species has been reported to produce oxidative exoenzymes that mobilize organic N, such as lignin and humic acids in response to low N availability [61]–[65]. Similar conditions are prevalent in mine reclamation sites, which may select for ECM fungi that are better nutrient scavengers; ecological filtering or natural selection favors physiological adaptations that allow mycorrhizal symbionts to utilize limiting nitrogen from organic resources [66].

*Cenococcum* was also very abundant on the parent trees, and sparse on the 5-month-old seedlings. This genera has short-ranging dark mycelia with thick, melanized cell walls [67], which may not be as quick to disperse via mycelia from established trees. This ECM has been reported to colonize weakened trees associated with declining oak forests [68],[69] and has been shown to increase in abundance in the absence of better competitors [70]. However, in this study, *Cenococcum* was not exclusive to declining trees and coexisted with *Cortinarius* on established trees. Further, seven-year-old chestnut trees colonized by either *Cenococcum* or *Cortinarius* were similar in height, and significantly taller than trees colonized with less than 25% ECM [39]. Corcobado et al. [47] also reported that *C. geophilum* was not associated with tree decline or root disease, but rather corresponded to dry soil conditions, which complements Pigott's [71] characterization of *Cenococcum* as having superior tolerance to water stress. Therefore, its presence may have been selected by drought conditions that were identified within Ohio throughout the time of this survey [72]. *Cenococcum*'s melanized morphology is thought to protect roots against desiccation and fluctuating temperatures [73], which may aid in mitigating UV radiation, heavy metals and water stress [74]–[76].

Other ECM species such as *S. areolatum* and *R. pectinatoides* were evenly shared between both chestnut parent trees and seedlings representing other important inoculum sources to incoming seedlings. Previous work on abandoned coal mine soils also showed that chestnut seedlings growing among established pines (*Pinus* *virginiana*) shared root colonization by *Scleroderma*, which were also documented with higher growth and survival [30]. Although disease did not influence ECM community differences, the age of the chestnut host did. For example, species such as *Cenococcum*, *C.* *parvannulatus*, *R.* *cerolens* were not as common on the five-month-old seedlings, yet prevalent on the parent trees. In contrast, species such *Hebeloma* sp., *Inocybe* sp. and *Thelephora* sp. were primarily associated with the five-month-old seedlings. In comparison, these species are not cord forming and may come with a lower carbon cost to the establishing seedling, which may have been an efficient strategy in terms of carbon expenditure than longer distance, thick hyphal forming morphologies [57]. Species such as *Cortinarius* sp. 4, *Helotiales*, *Laccaria* sp., *Lactarius* sp., *Oidiodendron* sp. were rare in our sampling, therefore, the preferential age of host could not be determined.

Despite the initial inoculation of *Pisolithus tinctorius* in the field nursery, this fungus was not sampled in this study. Though this fungus readily forms ECM with chestnut [30], this species tends to be a poor competitor and quickly becomes displaced after field planting [38]. Bauman et al. [77] found that ECM species such as *Hebeloma crustuliniforme*, *Laccaria bicolor*, *Scleroderma polyrhizum*, *Amanita rubescens*, *Suillus luteus* also did not maintain their mycorrhizal association with chestnut seedlings after 12 months in the field. However when compared to non-inoculated control seedlings, this initial inoculation contributed significantly to the survival of chestnut after outplanting. Further, these species did not interfere with colonization from ECM species present in field soils. Therefore, ecological specificity can be a significant driving factor determining host specificity and the beneficial status of ECM colonization in the field [78]. However, despite the short-term persistence of fungal inoculum, these species play an important role in mitigating seedling transplant shock during the first few months of establishment.

## 5. Conclusion

In summary, early successional habitats are dynamic; ECM colonization is influenced by tree development and may facilitate the survival of other native hardwoods like oak, that associate with chestnut mycorrhiza as this stand develops [32],[79]. ECM colonization and inoculum potential were negatively impacted by disease dieback, but blight had no effect on ECM community composition or select for species more tolerant to reduced photosynthate. Further, diseased trees did provide inoculum, however, did not confer benefits such as greater survival and larger seedlings as did healthy neighbors. Tree mortality in the restoration plots caused a decrease in inoculum potential, however, may still contribute ecological benefits such as organic input, decomposition and nutrient cycling [80],[81], which may aid in soil development in mined and reclaimed sites. Further, mortality caused by blight may help diversify the stand and encourage the establishment of other hardwood species that may recruit into the site. Chestnut seedlings that were colonized by the most abundant ECM genus, *Cortinarius*, displayed an increased in nitrogen acquisition. We hypothesize that *Cenococcum*, also abundant on trees, may play a role in water relations while increasing soil carbon stored as recalcitrant fungal litter [82]. Differing plant-fungal pairings can result in significant variations in host response [83] with the selection of symbionts driven by both the soil environment and limiting resources, which, in turn, drives the succession of fungal communities within recovering soils.
